# Effectiveness of Deferred Combined Androgen Blockade Therapy Predicts Efficacy in Abiraterone Acetate Treated Metastatic Castration-Resistant Prostate Cancer Patients after Docetaxel

**DOI:** 10.3389/fphar.2017.00836

**Published:** 2017-11-22

**Authors:** Jian-Ri Li, Kun-Yuan Chiu, Shian-Shiang Wang, Cheng-Kuang Yang, Chuan-Shu Chen, Hao-Chung Ho, Chi-Feng Hung, Chen-Li Cheng, Chi-Rei Yang, Cheng-Che Chen, Shu-Chi Wang, Chia-Yen Lin, Chao-Hsiang Chang, Chiann-Yi Hsu, Yen-Chuan Ou

**Affiliations:** ^1^Division of Urology, Department of Surgery, Taichung Veterans General Hospital, Taichung, Taiwan; ^2^Institute of Medicine, Chung Sang Medical University, Taichung, Taiwan; ^3^Department of Medicine and Nursing, Hungkuang University, Taichung, Taiwan; ^4^Department of Applied Chemistry, National Chi Nan University, Nantou, Taiwan; ^5^Department of Urology, China Medical University Hospital, Taichung, Taiwan; ^6^Department of Medical Research, Taichung Veterans General Hospital, Taichung, Taiwan; ^7^Tung's Taichung MetroHarbor Hospital, Taichung, Taiwan

**Keywords:** abiraterone acetate, androgen deprivation therapy, castration-resistant prostate cancer, deferred combined androgen blockade, docetaxel

## Abstract

**Introduction:** Conventional anti-androgen regimens were widely used as an initiation or combined androgen blockade (CAB) therapy in advanced prostate cancer patients. Currently, new androgen pathway inhibitors such as abiraterone acetate (AA) and enzalutamide had been proven effective in metastatic castration resistant prostate cancer. In this study, we attempt to analyze the role of conventional anti-androgen drugs as deferred CAB therapy in castration-resistant prostate cancer patients.

**Materials and Methods:** From 2012 to 2017, 48 metastatic castration-resistant prostate cancer (CRPC) patients who received sequential treatments with primary androgen blockade, oral anti-androgen regimens, and docetaxel followed by AA treatment were included. We defined effective deferred CAB as any decline of PSA after add-on antiandrogen after CRPC. Patients were separated into effective and ineffective deferred CAB. Comparison between two groups in the first line androgen deprivation therapy duration, CRPC PSA level, pre-AA PSA level, chemotherapy dosages, duration, and patients progression free survival and overall survival after AA treatment were analyzed.

**Results:** Twenty-three patients (47.9%) achieved PSA decline after deferred CAB. Among total 48 patients, 24 patients experienced PSA decline more than 50% after AA treatment. The median PSA progression-free survival and overall survival after AA treatment in the total cohort of 48 patients were 4.4 and 24.3 months, respectively. The effective deferred CAB group showed significantly lower PSA level, lower percentage of PSA progression, higher total follow-up duration, higher percentage of surviving patients, better progression free survival, and overall survival estimate after AA treatment. Of the eight variables analyzed, effectiveness in deferred CAB showed positive association to progression free survival (HR 0.29, 95% CI 0.12–0.67, *p* = 0.004) and overall survival (HR 0.24, 95% CI 0.07–0.81, *p* = 0.022). First line androgen deprivation therapy (ADT) duration also showed positive association to overall survival (HR 0.95, 95% CI 0.91–0.99, *p* = 0.023).

**Conclusions:** Effectiveness of deferred CAB therapy was positively associated with progression free survival and overall survival of AA treatment after docetaxel. It can be used as a pre-treatment predictor.

## Introduction

Sequential drugs with androgen deprivation therapies, chemotherapies, and androgen receptor signaling inhibitors had been proven as the mainstay of treatment in metastatic prostate cancer (Mukherji et al., 2014; Lorente et al., 2015). Abiraterone acetate (AA) and enzalutamide were two of the newly developed regimens which can prolong overall survival in metastatic castration resistant prostate cancer (MCRPC) patients (Fizazi et al., 2012; Scher et al., 2012). Currently, both regimens had been proven effective in chemo-naïve or post-chemotherapy settings (Fizazi et al., 2012; Scher et al., 2012; Beer et al., 2014; Ryan et al., 2015). Both STRIVE and TERRAIN trials showed enzalutamide was superior in progression free survival in MCRPC patients (Penson et al., 2016; Shore et al., 2016). In the recent European Association of Urology (EAU) guidelines, there was no role of conventional anti-androgens while castration-resistant prostate cancer (CRPC) developed (Cornford et al., 2017). However, before AA and enzalutamide launched, anti-androgen regimens, such as cyproterone, bicalutamide, and flutamide were applied to extend biochemical control with small benefit in survival. The median response duration ranged from 4.2 to 11 months with the use of bicalutamide or flutamide (Fosså et al., 2001; Fujii et al., 2006; Nishimura et al., 2007). Besides, Kijima et al. reported PSA response after deferred combined androgen blockade (CAB) therapy using bicalutamide could be an indicator to predict subsequent treatment outcome (Kijima et al., 2012). Therefore, we conducted a clinical investigation of AA in MCRPC patients after chemotherapy and validated the association between AA treatment efficacy and deferred CAB effectiveness.

## Materials and methods

### Patients

This retrospectively chart-review study was conducted to analyze MCRPC patients after chemotherapy using AA between 2012 and 2017. Total 93 consecutive patients were identified, and 10 were excluded because of incomplete data or loss of follow-up; another 35 patients were excluded because of not eligible for deferred CAB. Deferred CAB therapy was defined as add-on anti-androgen regimens after CRPC. Among 48 included patients, the first line ADT used were orchiectomy in 6, and LH-RH agonist in 42. The retrospective chart review protocol was approved and certified by the institute review board of Taichung Veterans General Hospital, number CE13240A-2 and CE13240-3. All patients received AA 1,000 mg with prednisolone 5 or 10 mg per day and standard of care in pain control or adverse events management during therapy.

### Study assessment

**Figure 1:**
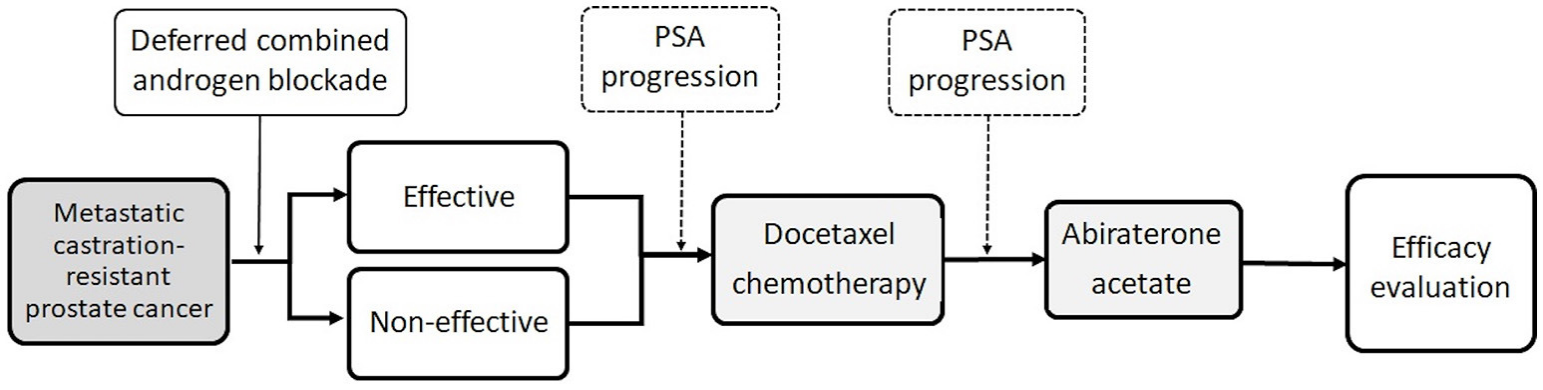
Flow chart demonstration of the management sequence.

Figure 1 demonstrated the management sequence during this study. The primary patient characteristics were age at start of AA treatment, serum PSA level while diagnosed MCRPC, PSA level while beginning of chemotherapy, PSA level while beginning of AA treatment, first-line chemotherapy cycles, first-line ADT therapy duration, chemotherapy duration, total follow-up duration, percentage of progression disease after AA treatment, and survival status.

Definition of deferred CAB efficacy was any PSA decline after add-on anti-androgen regimes after CRPC. All patients were then divided into the deferred CAB effective and ineffective groups. The chemotherapy cycles were defined according to the standard 3-week docetaxel treatment. In our practice, 2- and 4-week courses of chemotherapy were performed. We transferred the 2-week cycles into standard 3-week cycle counts. PSA progression was the only tool in the diagnosis of CRPC and AA treatment failure. PSA progression was defined according to the Prostate Cancer Working Group second publication (PCWG3) criteria (Scher et al., 2016). Although bone scans and clinical evaluation were performed in every patient, we still excluded both as monitoring end-point factors.

### Statistical analysis

The differences between continuous values were analyzed by Mann-Whitney U and Fisher's exact test *t*-test for continuous variables. Chi-square test was used for categorical variables. The progression free survival and overall survival curves were plotted by the Kaplan–Meier method. Univariate and multivariate Cox hazard regression was used to estimate the hazard ratio (HR) and 95% confidence interval (CI) for association between variables and progression-free and overall survival. All the statistical analyses were performed using SAS software version 9.2 (SAS Institute, Inc., Cary, NC, USA).

## Result

**Figure 2:**
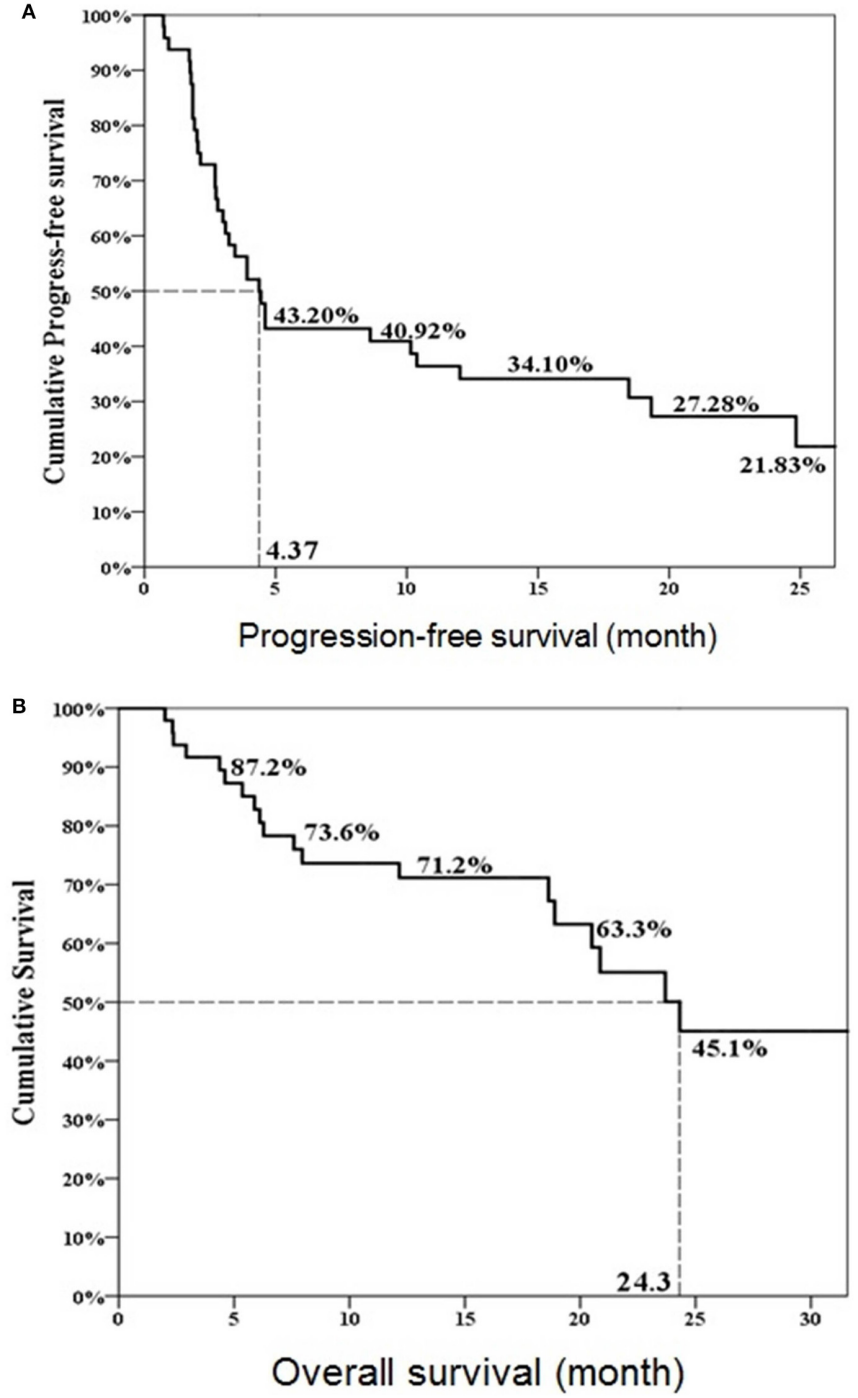
Progression-free survival **(A)** and overall survival **(B)** in total 48 patients.

**Figure 3:**
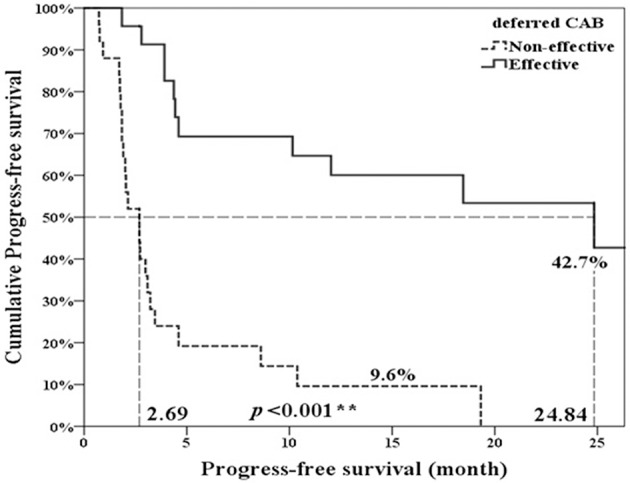
Progression-free survival comparison between ineffective and effective deferred CAB groups. **p < 0.01.

**Figure 4:**
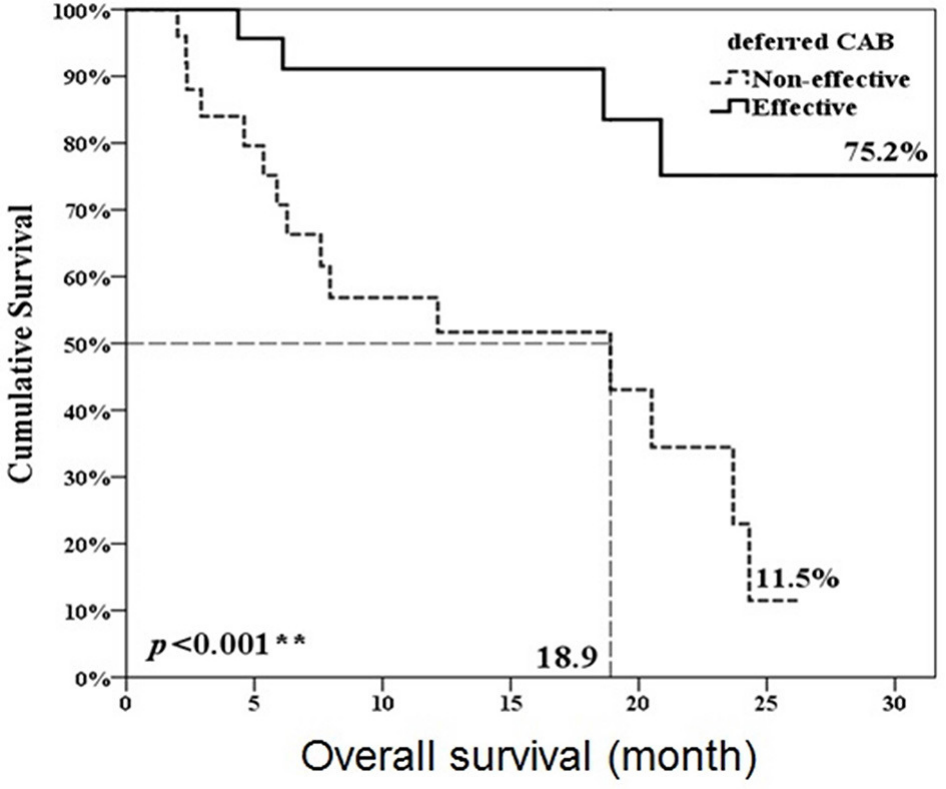
Overall survival comparison between ineffective and effective deferred CAB groups. **p < 0.01.

Twenty-three patients (47.9%, 23/48) met the criteria of effective deferred CAB. Table 1 illustrated the basic characteristics of the effective and ineffective deferred CAB groups. Among total 48 patients, 24 (50%) reached a PSA decline more than 50%. There were no significant differences in diagnosis age, start of ADT age, AA treatment age, percentage of previous radical prostatectomy, or therapeutic radiation therapy, pre-chemotherapy CRPC duration, chemotherapy cycles, chemotherapy duration, 1st line ADT duration, CRPC PSA and pre-AA PSA between two groups. The lowest PSA after AA, the percentage of PSA progression and the percentage of patient death were significantly lower in the effective deferred CAB group. The median follow-up duration among 48 patients was 17 months and it was significantly longer in the effective deferred CAB group. Among total 48 patients, the median progression-free survival was 4.4 months and the median overall survival was 24.3 months (Figure 2). Between two groups, the median progression-free survival and overall survival were significantly longer in the effective deferred CAB group than in the ineffective group (24.3 vs. 2.7 months; not reach median vs. 18.9 months, respectively, Figures 3, 4).

**Table 1:** Basic characteristics of deferred CAB effective and ineffective.

	**Total (*n* = 48)**	**deferred CAB**		***p*-value**
		**Non-effective (*n* = 25)**	**Effective (*n* = 23)**	
Diagnosis Age (years)	65 (60.3–72.8)	65 (60.5–75)	64 (60–72)	0.542
AA Age (years)	71.5 (64–79)	72 (64–79)	69 (64–79)	0.942
Initial PSA (*n* = 22 vs. 19)	198.9 (32–446)	68.9 (30–610.5)	293 (101–430)	0.308
Start ADT age (years)	66 (61.3–73)	66 (60.5–75)	65 (62–73)	0.664
Pre-chemo CRPC duration (month)	4.9 (1.6–11.8)	4.2 (1.8–6.9)	6.5 (1.6–17.1)	0.146
Chemo duration (month)	17.8 (8.6–31.9)	17.6 (8.1–29.7)	18.8 (10.7–32.1)	0.718
1st ADT duration (month)	21.4 (11.7–39.7)	18.7 (9.1–31.4)	31.2 (15.6–48.2)	0.157
Chemo cycle	7 (3.1–15.5)	7 (3.2–12)	7 (3–18)	0.740
Pre-chemo PSA	17.0 (5.8–49.5)	20.3 (7.2–71)	12.5 (4–36.1)	0.183
CRPC PSA	4.6 (2.7–10.2)	5.1 (2.7–14.3)	3.5 (2.7–9.4)	0.613
Pre-AA PSA	42.7 (18.9–167.8)	138 (16.6–619)	34.7 (20.8–96)	0.071
Best PSA after AA	17.6 (4.4–360.5)	104 (18.3–672)	4.8 (0.2–16.1)	<0.001^**^
Progression disease				0.002^**^
No	14 (29.2%)	2 (8.0%)	12 (52.2%)	
Yes	34 (70.8%)	23 (92.0%)	11 (47.8%)	
Total follow-up period (month)	17.0 (6.2–22.9)	8.0 (4.3–18.1)	18.6 (16.8–25.7)	0.002^**^
Survive				0.017^*^
Alive	28 (58.3%)	10 (40.0%)	18 (78.3%)	
Death	20 (41.7%)	15 (60.0%)	5 (21.7%)	
AA treatment effectiveness				<0.001^**^
Ineffective	24 (50.0%)	21 (84.0%)	3 (13.0%)	
Effective	24 (50.0%)	4 (16.0%)	20 (87.0%)	

Chi-square test. Mann-Whitney test.

* p < 0.05,

** *p < 0.01*.

*Continuous data were expressed median and IQR*.

*Categorical data were expressed number and percentage*.

There were 43 patients (89.6%) receiving bicalutamide 50 mg per day as deferred CAB therapy. Cyproterone was applied in 11 patients (22.9%), diethylstilbestrol in 9 (18.8%), and ketoconazole in 2 (4.2%). There were 15 (31.3%) patients having at least 2 anti-androgen treatments.

In univariate Cox regression analysis, effective deferred CAB and first-line ADT duration showed positive association to better progression free survival; pre-AA PSA showed a negative association to longer progression-free survival. After multivariate adjustment, only effective deferred CAB reached a statistical significance with association with PSA progression-free survival after AA treatment (HR 0.29, 95% CI 0.12–0.67, *p* = 0.004) (Table 2). In the analysis of overall survival, both effective deferred CAB and 1st line ADT duration reached the statistical significance (HR = 0.24, 95% CI 0.07–0.81, *p* = 0.022; HR 0.95, 95% CI 0.91–0.99, *p* = 0.023, respectively) (Table 3).

**Table 2:** Predictive variables of progression-free survival.

	**Univariate analysis**			**Multivariate analysis**		
	**HR**	**95%CI**	***p*-value**	**HR**	**95%CI**	***p*-value**
Deferred CAB						
Ineffective		ref.				
Effective	0.18	(0.08–0.39)	<0.001^**^	0.29	(0.12–0.67)	0.004^**^
Diagnosis Age	1.00	(0.96–1.04)	0.939			
AA Age	0.99	(0.95–1.02)	0.493			
Initial PSA	1.00	(1.00–1.00)	0.578			
Start ADT age	1.00	(0.96–1.04)	0.931			
Chemo duration (month)	0.99	(0.97–1.01)	0.348			
1st ADT duration (month)	0.98	(0.96–0.997)	0.025^*^	0.98	(0.96–1.00)	0.131
Chemo cycle	0.99	(0.95–1.04)	0.800			
Pre-chemo PSA	1.001	(0.9989–1.003)	0.321			
CRPC PSA	1.003	(0.997–1.009)	0.352			
Pre-AA PSA	1.001	(1.000–1.002)	0.002^**^	1.00	(1.00–1.00)	0.226

Cox regression. HR, Hazard Ratio. Adjusted for Deferred CAB, 1st ADT duration (month) and Pre-AA PSA.

* p < 0.05,

** *p < 0.01*.

**Table 3:** Predictive variables of overall survival.

	**Univariate analysis**			**Multivariate analysis**		
	**HR**	**95%CI**	***p*-value**	**HR**	**95%CI**	***p*-value**
Deferred CAB						
Ineffective		ref.			ref.	
Effective	0.17	(0.06–0.52)	0.002^**^	0.24	(0.07–0.81)	0.022^*^
Diagnosis Age	1.07	(1.01–1.14)	0.014			
AA Age	1.04	(0.98–1.10)	0.193			
Initial PSA	1.00	(1.00–1.00)	0.867			
Start ADT age	1.07	(1.01–1.14)	0.023^*^	1.06	(0.99–1.14)	0.115
Chemo duration (month)	0.98	(0.95–1.02)	0.284			
1st ADT duration (month)	0.95	(0.92–0.99)	0.006^**^	0.95	(0.91–0.99)	0.023^*^
Chemo cycle	0.96	(0.89–1.02)	0.198			
Pre-chemo PSA	1.005	(1.002–1.007)	0.001^**^	1.00	(1.00–1.01)	0.823
CRPC PSA	1.007	(1.001–1.013)	0.024^*^	1.00	(0.99–1.01)	0.677
Pre-AA PSA	1.001	(1.0002–1.002)	0.021^*^	1.00	(1.00–1.00)	0.366

Cox regression. HR, Hazard Ratio. Adjusted for Deferred CAB, Start ADT age, 1^st^ ADT duration (month), Pre-chemo PSA, CRPC PSA and Pre-AA PSA.

* p < 0.05,

** *p < 0.01*.

## Discussion

Our study was the first to identify the association between hormone manipulation after CRPC and AA treatment efficacy. This finding also implicated the possible role of conventional anti-androgen regimens in the current sequential treatments for prostate cancer.

Chi et al. reported a cumulative scoring system with six independent factors including serum lactate dehydrogenase (LDH), Eastern Cooperative Oncology Group Performance Status (ECOG PS), liver metastases, serum albumin, serum alkaline phosphatase (ALP) and first line ADT duration which can predict AA treatment efficacy (Chi et al., 2016). Their results implicated effectiveness of prior androgen receptor (AR) targeting therapy can indicate subsequent second line AR targeting treatment efficacy because the sequential treatments were both focusing on AR. Our previous data also showed similar AR targeting prediction effect (Li et al., 2017). On the other hand, Flaig et al. reported a reverse proof of the AR targeting. Their prospective trial recruited suboptimal PSA response patients in the 1st line ADT and only 13% PSA response rate was made after AA treatment (Flaig et al., 2017). In this study, we not only echoed our previous results, but also a new association between effective add-on anti-androgens after CRPC and the efficacy of subsequent second line anti-androgen therapy.

Kijima et al. revealed a predicting role using bicalutamide after CRPC in the subsequent AR targeting therapy and chemotherapy (Kijima et al., 2012). Their findings indicated the short period between CRPC and chemotherapy may contain a predicting model which guided clinicians to go AR targeting therapy or not. Their hypothesis came from many effective clinical reports using deferred CAB. Although the genetic variation after CRPC had been identified for more than 2 decades, in this new treatment bursting era, we still need more clinical observations from the patients to find out a reasonable treatment strategy.

In our study, bicalutamide was the most common used anti-androgen regimen during deferred CAB. Although 31.3% patients received at least 2 anti-androgens after CRPC, the overall median treatment duration was only 4.9 months. We think this was because of the clinician-patients PSA anxiety during treatment shifting in order not to compromise standard treatments. In most of time, clinicians would rather shift to next line chemotherapy once frequent PSA survey showed elevating.

Our overall progression-free survival was only 4.4 months which was shorted than 8.5 months in the COU-AA-301 trial (Fizazi et al., 2012). The median overall survival was 24.3 months and the PSA response rate was 50% which showed better in indirect comparison with the previous trial. These variant results may come from our retrospective study design and small study numbers.

Currently, the EAU guideline had no recommendation of the conventional anti-androgen regimens use in the sequential treatment of metastatic prostate cancer except combination with LH-RH agonists in the beginning to compress the testosterone surge (Cornford et al., 2017). From the results of our study, the applications of these conventional anti-androgens seemed to be directed to a new way. However, we still need more prospective data to prove this hypothesis.

There were still several limitations in this retrospective study. First, as a major tool to measure progression, the PSA check-up schedules were not well controlled due to the retrospective setting. Second, it is now considered PCWG3 consensus as a recommended tool to evaluate CRPC studies which was not applied well in our real world practice. Third, the anti-androgens used in the present study were too diverse to show an identical treatment effect. Fourth, our sample size was too small and a lot of original data were excluded which may lead to low statistical power and selection bias.

## Conclusion

Our data showed a positive association between effectiveness of deferred CAB after CRPC and subsequent AA treatment efficacy after docetaxel. This pre-AA treatment prognostic marker could help clinicians in patient planning and provided conventional anti-androgens a new application direction.

## Author contributions

Study design: J-RL, C-HC, Y-CO. Data collecting: K-YC, S-SW, C-KY, C-SC, H-CH, C-FH, C-RY, C-CC, S-CW, C-YL. Data analysis: J-RL, C-YH. Manuscript writing: J-RL. Modification: C-LC, Y-CO.

### Conflict of interest statement

The authors declare that the research was conducted in the absence of any commercial or financial relationships that could be construed as a potential conflict of interest.

## References

[B1] Beer T. M. Armstrong A. J. Rathkopf D. E. Loriot Y. Sternberg C. N. Higano C. S. (2014). Enzalutamide in metastatic prostate cancer before chemotherapy. N. Engl. J. Med. 371, 424–433. 10.1056/NEJMoa1405095 24881730 PMC4418931

[B2] Chi K. N. Kheoh T. Ryan C. J. Molina A. Bellmunt J. Vogelzang N. J.. (2016). A prognostic index model for predicting overall survival in patients with metastatic castration-resistant prostate cancer treated with abiraterone acetate after docetaxel. Ann. Oncol. 27, 454–460. 10.1093/annonc/mdv594 26685010 PMC4769990

[B3] Cornford P. Bellmunt J. Bolla M. Briers E. De Santis M. Gross T.. (2017). EAU-ESTRO-SIOG guidelines on prostate cancer. Part II: Treatment of Relapsing, Metastatic, and Castration-Resistant Prostate Cancer. Eur. Urol. 71, 630–642. 10.1016/j.eururo.2016.08.002 27591931

[B4] Fizazi K. Scher H. I. Molina A. Logothetis C. J. Chi K. N. Jones R. J.. (2012). Abiraterone acetate for treatment of metastatic castration-resistant prostate cancer: final overall survival analysis of the COU-AA-301 randomised, double-blind, placebo-controlled phase 3 study. Lancet Oncol. 13, 983–992. 10.1016/S1470-2045(12)70379-0 22995653

[B5] Flaig T. W. Plets M. Hussain M. H. Agarwal N. Mitsiades N. Deshpande H. A.. (2017). Abiraterone acetate for metastatic prostate cancer in patients with suboptimal biochemical response to hormone induction. JAMA Oncol. 10.1001/jamaoncol.2017.0231 28358937 PMC5824213

[B6] Fosså S. D. Slee P. H. Brausi M. Horenblas S. Hall R. R. Hetherington J. W. (2001). Flutamide vs. prednisone in patients with prostate cancer symptomatically progressing after androgen-ablative therapy: a phase III study of the European organization for research and treatment of cancer genitourinary group. J. Clin. Oncol. 19, 62–71. 10.1200/JCO.2001.19.1.62 11134196

[B7] Fujii Y. Kawakami S. Masuda H. Kobayashi T. Hyochi N. Kageyama Y.. (2006). Deferred combined androgen blockade therapy using bicalutamide in patients with hormone-refractory prostate cancer during androgen deprivation monotherapy. BJU Int. 97, 1184–1189. 10.1111/j.1464-410X.2006.06149.x 16686709

[B8] Kijima T. Fujii Y. Yokoyama M. Ishioka J. Matsuoka Y. Numao N.. (2012). Prostate-specific antigen response to deferred combined androgen blockade therapy using bicalutamide predicts survival after subsequent oestrogen and docetaxel therapies in patients with castration-resistant prostate cancer. BJU Int. 110, 1149–1155. 10.1111/j.1464-410X.2012.10959.x 22369348

[B9] Li J. R. Wang S. S. Yang C. K. Chen C. S. Ho H. C. Chiu K. Y.. (2017). First line androgen deprivation therapy duration is associated with the efficacy of abiraterone acetate treated metastatic castration-resistant prostate cancer after docetaxel. Front. Pharmacol. 8:55. 10.3389/fphar.2017.00055 28243202 PMC5304424

[B10] Lorente D. Mateo J. Perez-Lopez R. de Bono J. S. Attard G. (2015). Sequencing of agents in castration-resistant prostate cancer. Lancet Oncol. 16, e279–e292. 10.1016/S1470-2045(15)70033-1 26065613

[B11] Mukherji D. Omlin A. Pezaro C. Shamseddine A. de Bono J. (2014). Metastatic castration-resistant prostate cancer (CRPC): preclinical and clinical evidence for the sequential use of novel therapeutics. Cancer Metastasis Rev. 33, 555–566. 10.1007/s10555-013-9473-1 24452758

[B12] Nishimura K. Arichi N. Tokugawa S. Yoshioka I. Kishikawa H. Ichikawa Y. (2007). Effects of flutamide as a second-line agent for maximum androgen blockade of hormone refractory prostate cancer. Int. J. Urol. 14, 264–267. 10.1111/j.1442-2042.2007.01681.x 17430272

[B13] Penson D. F. Armstrong A. J. Concepcion R. Agarwal N. Olsson C. Karsh L. (2016). Enzalutamide vs. bicalutamide in castration-resistant prostate cancer: the STRIVE trial. J. Clin. Oncol. 34, 2098–2106. 10.1200/JCO.2015.64.9285 26811535

[B14] Ryan C. J. Smith M. R. Fizazi K. Saad F. Mulders P. F. Sternberg C. N. (2015). Abiraterone acetate plus prednisone vs. placebo plus prednisone in chemotherapy-naive men with metastatic castration-resistant prostate cancer (COU-AA-302): final overall survival analysis of a randomised, double-blind, placebo-controlled phase 3 study. Lancet Oncol. 16, 152–160. 10.1016/S1470-2045(14)71205-7 25601341

[B15] Scher H. I. Fizazi K. Saad F. Taplin M. E. Sternberg C. N. Miller K.. (2012). Increased survival with enzalutamide in prostate cancer after chemotherapy. N. Engl. J. Med. 367, 1187–1197. 10.1056/NEJMoa1207506 22894553

[B16] Scher H. I. Morris M. J. Stadler W. M. Higano C. Basch E. Fizazi K.. (2016). Trial design and objectives for castration-resistant prostate cancer: updated recommendations from the prostate cancer clinical trials working group 3. J. Clin. Oncol. 34, 1402–1418. 10.1200/JCO.2015.64.2702 26903579 PMC4872347

[B17] Shore N. D. Chowdhury S. Villers A. Klotz L. Siemens D. R. Phung D. (2016). Efficacy and safety of enzalutamide vs. bicalutamide for patients with metastatic prostate cancer (TERRAIN): a randomised, double-blind, phase 2 study. Lancet Oncol. 17, 153–163. 10.1016/S1470-2045(15)00518-5 26774508

